# Conservative management following patellar dislocation: a level I systematic review

**DOI:** 10.1186/s13018-023-03867-6

**Published:** 2023-05-30

**Authors:** Gustavo Wickert Flores, Deise Ferreira de Oliveira, Ana Paula Silveira Ramos, Luciana Sayuri Sanada, Filippo Migliorini, Nicola Maffulli, Rodrigo Okubo

**Affiliations:** 1 Department of Physiotherapy, University of the State of Santa Catarina, Florianópolis, SC Brazil; 2 Physiotherapy Department, University of South of Santa Catarina (Unisul), Florianópolis, SC Brazil; 3 Department of Orthopaedic, Trauma, and Reconstructive Surgery, RWTH University Hospital, Pauwelsstraße 30, 52074 Aachen, Germany; 4 Department of Medicine, Surgery and Dentistry, University of Salerno, 84081 Baronissi, SA Italy; 5 School of Pharmacy and Bioengineering, Keele University Faculty of Medicine, Stoke on Trent, ST4 7QB England, UK; 6 Barts and the London School of Medicine and Dentistry, Centre for Sports and Exercise Medicine, Mile End Hospital, Queen Mary University of London, London, E1 4DG England, UK; 7 Department of Orthopaedics and Trauma Surgery, Academic Hospital of Bolzano (SABES-ASDAA), 39100 Bolzano, Italy

**Keywords:** Patella, Instability, Dislocation, Physiotherapy, Rehabilitation

## Abstract

**Background:**

Patellar instability is a common and disabling clinical condition. Treatment of acute primary patellar dislocation aims to reduce the risk of recurrence or painful subluxation and improve function. However, the actual clinical efficacy of any management modality following an acute dislocation has never been demonstrated in prospective or retrospective studies, and the optimal way in which the various management modalities should be used is at best unclear.

**Methods:**

A search was conducted in PubMed, Bireme and Embase databases. Inclusion criteria followed the acronym PICOS, (P) subjects with patellar instability, (I) therapeutic interventions, (C) placebo or control or surgical treatments, (O) rate of dislocations and function, and (S) clinical trials. The Medical Subject Headings (MeSH) terms used were: ((“patellar instability”) OR (“patellar dislocation”)) AND ((physiotherapy) OR (rehabilitation) OR (“conservative treatment”) OR (therapy) OR (therapeutic)). The risk of bias was analysed using the PeDRO scale.

**Results:**

Seven randomized controlled trials including 282 patients were considered. The quality of studies detailing the results of conservative treatment was higher than that of surgical procedures, but all studies have relatively low methodological quality. Four studies compared physiotherapeutic interventions with surgical procedures, and three studies compared conservative intervention techniques.

**Conclusion:**

An unstructured lower limb physical therapy programme evidences similar outcomes to specific exercises. Surgical management is associated with a lower rate of re-dislocation; however, whether surgery produces greater functional outcomes than conservative management is still unclear. The use of a knee brace with a limited range of motion, stretching and neuromuscular exercises are the most commonly recommended physiotherapy methodologies.

## Introduction

Patients with patellofemoral instability exhibit abnormal patellar tracking over the femoral trochlea during motion [1, 2] and report discomfort during prolonged knee flexion or during sports activities [3–5]. Several risk factors predispose to patellofemoral instability, including patella alta, trochlear dysplasia, muscle imbalance, increased distance between the tibial tubercle (TT) and trochlear groove (TG), valgus, and femoral deformity, especially anteversion [6–9]. Patellar dislocation accounts for 2–3% of all knee injuries [10]. The incidence of patellar dislocation is between 2 and 77 per 100,000 people per year [11–14]. Furthermore, 61% of primary dislocations occur during sports activities and are mostly seen in teenagers and young adults [15, 16].

The management of patellar dislocation is controversial [17–19]. Following an acute episode of patellar dislocation, in patients without osteochondral damage or intraarticular loose bodies, conservative management could be undertaken [20]. Conservative management should provide rapid functional recovery and minimize the evolution to recurrent patellar dislocation [21–23]. The current literature emphasizes isometric quadriceps strengthening, specific strengthening of the vastus medialis obliquus, and progression to more dynamic exercises involving the core and gluteal muscles [23–25]. However, international consensus or guidelines on conservative management are lacking, and high-quality evidence is required [26]. Recent guidelines on first-time patellar dislocation cite that, despite the lack of rigorous clinical evidence, many reviews report opinions and recommendations derive from the expertise and experience of the authors [27]. This systematic review assessed the efficacy of conservative interventions for patients with patellar instability. This study investigates the outcomes of conservative and surgical management in adults with patellofemoral instability. The efficacy of conservative management in adults with patellofemoral instability is still controversial. About one-third of patients treated conservatively have activity limitations 6 months to 3 years following the patellar dislocation, even in the absence of re-dislocation. Surgical treatment is associated with a low rate of recidivism and good outcomes and levels of sport participation. However, whether surgery is associated with better outcomes than conservative management remains unclear.

## Methods

A systematic literature review was conducted according to the Preferred Reporting Items for Systematic Reviews and Meta-Analyses (PRISMA) statement [28, 29] and registered in PROSPERO (ID CRD42022370928).

### Search strategy and database

The literature search was performed by two authors (GWF and DFO) independently. The search keywords were determined through the acronym PICOS:

- P (population): adult patients (> 18 years old) with patellar instability
- I (intervention): conservative management
- C (comparator): placebo, control, surgical management
- O (outcomes): joint function, failures
- S (studies): Randomized controlled trials (RCTs) level I of evidence

The databases used were PubMed, Bireme, and Embase; the search took place on May 2023. The Medical Subject Headings (MeSH) terms in English used were: ((“patellar instability”) OR (“patellar dislocation”)) AND ((physiotherapy) OR (rehabilitation) OR (“conservative treatment”) OR (therapy) OR (therapeutic)).

### Eligibility

All the clinical studies investigating the outcome of conservative management of patellofemoral instability, such as physiotherapy treatment, rehabilitation, exercise, and immobilization, were included. The eligibility of study participants for each study was confirmed if they had a reported history of patellar dislocation, either primary or recurrent. Only RCT level I of evidence, according to the Oxford Centre of Evidence Based Medicine [30], were eligible. Studies that reported quantitative data joint function and the rate of failures were eligible. Articles in English, Spanish, Italian, and Portuguese were considered.

Comments, reviews, case reports, editorials, letters to the editor, and technical notes were not eligible. Studies missing quantitative data under the outcomes of interest were excluded. Two authors extracted data independently using Rayaan® Free Trial [31], and discrepancies were resolved by a third one (**).

### Assessment of methodological quality

The methodological quality assessment was conducted by two reviewers (GWF and APSR), using the PeDRO Scale [32]. Discrepancies were resolved by a third reviewer (**). This scale is a tool developed to measure the methodological quality of studies of physiotherapy interventions. It consists of 10 items plus selection criteria: (1) randomization of the sample; (2) concealed allocation; (3) initial comparability between groups; (4) all subjects blinded; (5) all therapists who administer therapy blinded; (6) all evaluators measuring key outcomes blinded; (7) adequacy of follow-up; (8) statistical analysis with intention to treat; (9) statistical comparison of results between groups; and (10) existence of specific measures and variability for at least one key result. These items are dichotomous, and each question is scored as 1 or 0 [32, 33]. The PeDRO Scale has been widely used in previous systematic reviews [34–37].

## Results

### Selection of studies

**Fig. 1:**
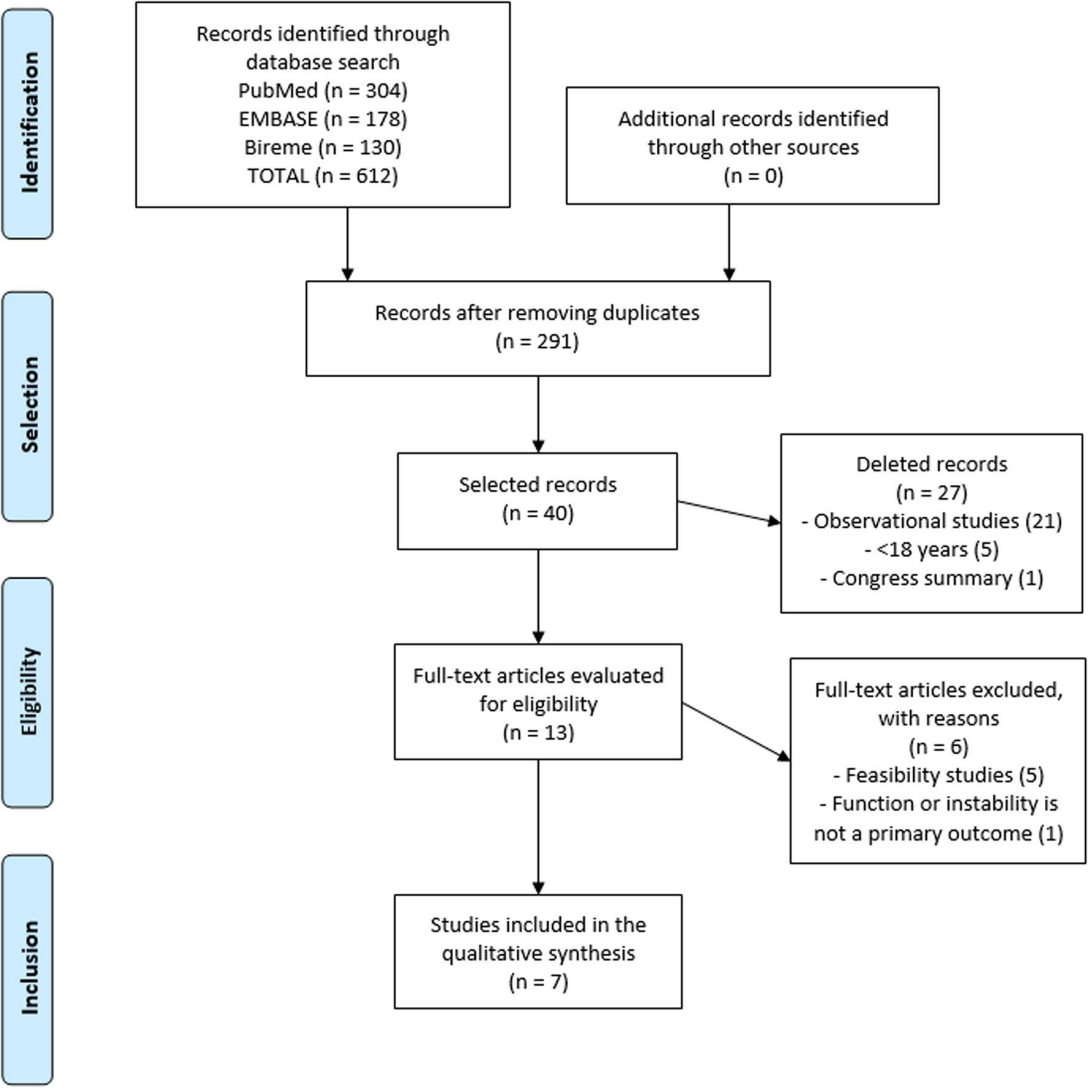
PRISMA flowchart of search in the literature

The literature search resulted in 612 articles: 130 in Bireme, 178 in Embase, and 304 in PubMed. After removing duplicates, 291 remained for consideration. After reading the titles, 40 abstracts remained. After this screening, 27 articles were excluded because they were observational studies (*n* = 21) or congress summaries (*n* = 1); therefore, 13 complete articles were selected for the reading stage. In the last stage, 6 articles were excluded since they were feasibility studies (*n* = 5) and function or instability was not a primary outcome (*n* = 1). Finally, seven articles were included in the present investigation (Fig. 1).

### Methodological quality

No study performed participants blinding, negatively impacting the final result. High between studies variability in the PeDRO scale was evidenced, with values ranging from seven to three points. Concluding, the average score of the PeDRO scale was 4.1 points, demonstrating the low quality of the methodology (Table 1).

**Table 1:** Analysis of the methodological quality of the selected studies—PeDRO (*n* = 7)

References	1	2	3	4	5	6	7	8	9	10	11	Total
Camanho et al. [38]	Yes	Yes	Yes	No	No	No	No	No	No	Yes	No	3
Bitar et al. [39]	Yes	Yes	No	No	No	No	No	Yes	No	Yes	Yes	4
Smith et al. [40]	Yes	Yes	Yes	Yes	No	No	Yes	No	Yes	Yes	Yes	7
Rood et al. [21]	Yes	Yes	No	No	No	No	Yes	Yes	No	Yes	Yes	5
Straume‑Nasheim et al. [41]	Yes	Yes	Yes	Yes	No	No	No	No	No	Yes	Yes	5
Honkonen et al. [42]	Yes	Yes	No	Yes	No	No	No	No	No	Yes	Yes	4
Petri et al. [20]	Yes	Yes	No	Yes	No	No	No	Yes	Yes	Yes	No	5

1. Eligibility criteria have been specified

2. Subjects were randomly assigned to groups

3. The distribution of subjects was blind

4. Initially, the groups were similar with regard to the most important prognostic indicators

5. All subjects participated blindly in the study

6. All physiotherapists who administered the therapy did so blindly

7. All evaluators who measured at least one key outcome did so blindly

8. Measurements of at least one key outcome were obtained in more than 85% of subjects initially assigned to groups

9. All subjects from whom outcome measurements were presented received the treatment or control condition as per the distribution or, when this was not the case, data analysis was performed for at least one of the key outcomes by “intent to treat”

10. The results of inter-group statistical comparisons were described for at least one key outcome

11. The study has both precision measures and variability measures for at least one key outcome

### Characteristics of the studies and participants

Data from 282 patients were retrieved (Table 2). Four studies compared physiotherapeutic interventions with surgical procedures [20, 38, 39, 41], and three studies compared conservative intervention techniques [21, 40, 42].

**Table 2:** Results of studies performing conservative strategies

References	Country	Objective	Sample (*n*)	Evaluation	Characteristics of the therapeutic intervention	Function	Recurrence of dislocations/instability	Other outcomes
Straume‑Næsheim et al. [41]	Norway	To compare surgery (reconstruction of the medial patellofemoral ligament (MPFL-R)) with active rehabilitation in the absence of anatomical changes such as risk factors	- 61 patients (surgery, *n* = 30, rehabilitation, *n* = 31)	- Patellar instability PISS and (BHS) - Function (PROMS): KOOS, Kujala, Cincinnati, Lysholm and Noyes Sport Activity (Baseline and 12 months)	- Home exercises and referral to physiotherapy - VMO strengthening training program, hamstring stretching and knee neuromuscular balance. McConnell patellar brace or bandage	- PROMs (*p* > 0.05) comparing surgery and rehabilitation	- Persistent instability (12 months): 13 (41.9%) rehabilitation, 2 (6.7%) surgery (RR 6.3 (95% CI 1.5–25.5)	- Anterior knee pain: 6 (20%) in the surgery group - Complex regional syndrome in the surgery group (1) - ROM without differences between groups at follow-up
Smith et al. [40]	UK	To compare VMO strengthening with general quadriceps strengthening after first patellar dislocation	- 50 patients - General quadriceps exercises (*n* = 25) - Specific VMO exercises (*n* = 25)	- Instability: Norwich Patellar Instability (NPI) - Function: Lysholm and Tegner Level of Activity - Isometric knee extension strength in various knee ROMs (baseline, 6 weeks, 6 months and 12 months	- Standard treatment was immobilization (3–4 weeks) in an extension splint, followed by physiotherapy - Exercise diary - The program was aimed at reducing pain, swelling and stiffness and increasing ROM and function. Exercises designed to strengthen or recruit VMO or the group-dependent quadriceps complex	- Tegner (12 months) in the general quadriceps exercise group compared to the VMO group (*p* = 0.04; 95% CI − 3.0 to 0.0) - Lysholm (*p* = 0.05)	- There was no statistically significant difference between groups for NPI - 2 episodes were observed (VMO group)	- There was no statistically significant difference between groups for isometric strength
Rood et al. [21]	Netherlands	To evaluate whether taping results in better functional results and patellar stability in the short term	- 18 patients - Bandage (*n* = 9) - Immobilization with plaster (*n* = 9)	- Function: Lysholm Knee Scoring Scale [18] was at 1, 6 and 12 weeks and at 1- and 5-year post-dislocation - Instability: Dislocation episodes - Circumference of the quadriceps	- 1st week post-dislocation fixed splint. Afterwards, peripatellar taping to avoid re-dislocation (allowing 30°–40° of Flexion) or plaster - Six weeks after dislocation, intensive quadriceps training (isometric and isotonic exercises)	- Lysholm after 6 weeks, with a mean of 58 for the tape group and 26 for the plaster group (*p* = 0.001) - After 12 weeks and 5 years, also favourable to tape (*p* = 0.02 and *p* = 0.008)	- No cases of recurrence	- Tape muscle hypotrophy was significantly lower (5.7 vs. 2.1 cm, *P* < 0.001)
Honkonen et al. [42]	Finland	To compare a patellar stabilizing and movement-restricting brace versus a non-articulated neoprene brace for the treatment of a first traumatic patellar dislocation	- 64 patients - Group A (*n* = 32) (patellar stabilizer brace and articulated for 0°–30° ROM only) - Group B (*n* = 32) (neoprene brace (no ROM restriction)	- Numbers of shifts - Pain (VAS) - ROM of the knee - Tegner Activity Scale - Score of Kujala - Muscular trophism of the quadriceps - Follow-up: 4 weeks, 3 months, 6 months, 12 months, 24 months and 36 months after the initial trauma	- Braces were used according to groups and both groups were advised: use of crutches for as long as necessary with full load as tolerated. All patients received similar physiotherapeutic instructions (exercise to strengthen the quadriceps muscles and lower limbs in closed kinetic chain)	- Kujala: Group A < B at 6 months (89.0 vs 93.6, [95% CI 1.07–8.14]; *p* = 0.01) - Tegner: No differences	- Group A was 34.4% (11/32). Group B, rate of 37.5% (12/32) (RD, − 3.1% [95% CI 26.6–20.3%]; *p* = 0.8) - Group A: 20/32 cases (62.5%) and B: 19/32 (59.4%) reported subjective joint instability symptoms	- ROM: Group A < B at 4 weeks (90° vs 115°; *p* < 0.001), at 3 months (125° vs 133°; *p* = 0.03), at 6 months (*p* > 0.05) - Hypotrophy: Group A > B at 4 weeks and 3 months (24/32 vs 16/32, *p* = 0.5) - VAS: No differences
Camanho et al. [38]	Brazil	To compare surgical (MPFL-R and femoral insertion) and conservative treatment for acute patellar dislocations	- 33 patients (acute patellar dislocation) - Conservative (*n* = 16) - Surgical (*n* = 17)	- Numbers of recurring shifts - Role: Kujala - Average follow-up of 40.4 months	- Conservative: immobilization (3 weeks) - Rehabilitation started after this period with exercises to strengthen the lower limbs (emphasis on VMO), stretching for the hamstrings and the articular retinaculum were only performed 1 month after the trauma or surgery. The treatment time varied between 2 and 4 months (without pain)	- Kujala: Conservative (average 69 points), surgical (average 90–92. 22% improvement)	- Dislocations: Conservative 8/16; Surgical no case reported	- N/a
Petri et al. [20]	Germany	To compare conservative and surgical “repair the tear” treatment in patients after first patellar dislocation	- 20 patients (acute patellar dislocation) - Conservative (*n* = 8) - Surgical (*n* = 12)	- Patellar instability severity score - Numbers of recurring shifts - Role: Kujala - Follow-up at 6, 12 and 24 months	- Conservative and surgical were treated with a ROM brace 0°–0°–60° + partial load 15 kg crutches (first 3 weeks) and 0°–0°–90° (3–6 weeks) - Progression to full pain-adapted therapy	- Kujala: Conservative vs operative—78.6 vs 80.3 after 6 months (*p* = 0.8), 79.9 vs 88.9 after 12 months (*p* = 0.2) and 81.3 vs 87.5 after 24 months (*p* = 0.3)	- Conservative: 37.5% (3/8) had been dislocated - Surgical: 16.7% (2/12) suffered dislocation within 24 months - *p* = 0.5	- N/a
Bitar et al. [39]	Brazil	To compare the results of surgical (MPFL-R) versus non-surgical treatment in the treatment of primary patellar dislocation	- 36 patients (acute patellar dislocation) - Conservative (*n* = 18) - Surgical (*n* = 18)	- Numbers of recurring shifts - Role: Kujala - Minimum follow-up of 24 months	- Use of extension brace for 3 weeks and physical therapy focusing on ROM and quadriceps strengthening. Isometric quadriceps exercises, analgesia, cryotherapy, and electrical stimulation. Weight bearing after 3 weeks. Afterward, exercises were increased to gain ROM and the ergometric bicycle without load was introduced. Initial proprioception and closed kinetic chain exercises were performed and gradually evolved. Target time of 16–24 weeks	- Kujala: Conservative (70.8) < compared with Surgical group (88.9; *p* = 0.001).—Surgery had a higher percentage of ''good/excellent results'' (71.43%) in the Kujala, compared with the conservative group (25.0%; *p* = 0.003)	The conservative group had > number of recurrences and subluxations (7 patients; 35% of cases), whereas there were no reports of recurrences or subluxations in the surgical group	- N/a

*PISS* Patellar Instability Severity Score, *BHS* Beighton Hypermobility Score, *PROMS* Patient Reported Outcome Measures, *KOOS* Knee injury and Osteoarthritis Outcome Score, *VMO* Vastus Medialis Oblique, *VAS* Visual Analog Scale, *N/a* not applicable

### Conservative intervention

All studies suggested the use of braces (with total or partial immobilization) for the initial period of the first 3 weeks. Concerning weight bearing, one RCT recommended progressive weight according to pain [42], and another investigation recommended 15 kg partial weight bearing for the first 3 weeks [20]. The conservative interventions implemented were: strengthening the quadriceps, in particular the vastus medialis muscle [38, 40, 41] and hamstring [38, 41], closed kinetic chain exercises [39, 42]; increasing proprioception and balance [39, 41].

Smith et al. [40] observed a statistical difference in the Lysholm knee score and Tegner Level of Activity score between general quadriceps and VM exercise groups at 12 months; however, there was no statistically or clinically significant difference for these measures during the first 12 months post-commencement of rehabilitation following patellar dislocation.

Honkonen et al. [42], in addition to quadriceps muscle strengthening exercises, closed kinetic chain lower limb, and full weight bearing as tolerated by pain for both groups, compared the efficacy of a patella-stabilizing, motion-restricting knee brace versus a neoprene non-hinged knee brace for the treatment of first-time traumatic patellar dislocation. Knee immobilization was associated with quadriceps muscle atrophy, more restricted knee ROM, and worse functional outcomes in the first 6 months after the injury. In another study, cylinder cast immobilization was compared to taping in terms of intensive training of isometric and isotonic exercises to strengthen quadriceps muscles. Both groups were allowed the full weight-bearing [21]. After 12 weeks and 5 years, the Lysholm Knee Scoring Scale was significantly better in the taping group.

### Conservative and surgical treatment

Four RCTs compared conservative versus surgical management [20, 38, 39, 41]. Surgical management included reconstruction of the medial patellofemoral ligament (MPFL-R) [38, 39, 41], femoral re-insertion [38] and “repair the tear” [20]. Surgical management evidenced greater functional results and a lower rate of recurrence of dislocations [38, 39]. Camanho et al. [38] reported a higher number of recurrent dislocations (8 patients) in the conservative treatment group compared to the surgical treatment group, which did not experience any relapses. In addition, the surgical management group obtained a better mean score on the Kujala test (92) than the conservative treatment group. Bitar et al. [39] showed that the surgical group presented a higher percentage of ‘‘good/excellent’’ results (71.43%) on the Kujala score when compared with the non-operative group (25.0%; *p* = 0.003). The non-operative group presented a large number of recurrences and subluxations (7 patients; 35% of cases), whereas no recurrences or subluxations occurred in the surgical group.

One study showed no differences in function between treatments, but more dislocations in patients managed non-operatively [41]. Straume‑Næsheim et al. [41] showed persistent patellar instability at 12 months in 13 (41.9%) controls, versus 2 (6.7%) in the surgical group (RR 6.3 (95% CI 1.5–25.5). The patients with persistent instability at 12 months did not score significantly lower on any of the PROMs compared to their stable peers, regardless of the study group.

One study showed no differences in function and dislocation rate between treatments [20]. Petri et al. [20] showed a mean Kujala score of the conservative vs operative treatment group of 78.6 vs 80.3 after 6 months (*p* = 0.8), 79.9 vs 88.9 after 12 months (*p* = 0.2), and 81.3 vs 87.5 after 24 months (*p* = 0.3). The re-dislocation rate after 24 months was 37.5% in the conservative group and 16.7% in the operative group (*p* = 0.4).

## Discussion

According to the main findings of the present systematic review of level I evidence, surgical management is associated with a lower rate of re-dislocation; however, whether surgery promotes greater functional outcomes than conservative management is still unclear. The use of a knee brace with a limited range of motion, stretching and neuromuscular exercises are the most commonly recommended methodologies of physiotherapy. In cases of primary patellar dislocation associated with large displaced osteochondral fractures (> 5 mm) or chondral shear fragments and/or complete VMO avulsion of the patellar insertion site, surgery is indicated [43]. However, the management of the patients who experienced traumatic patellar dislocation with no evidence of osteochondral injuries or intraarticular loose bodies is still controversial [17, 44, 45].

Straume‑Næsheim et al. [41] compared MPFL-R surgery with active rehabilitation in patients with recurrent patellofemoral instability. Patients with recurrent patellar dislocations have a six times greater risk of persistent patellar instability if treated with active rehabilitation alone, compared to active rehabilitation combined with MPFL-R, even in the absence of significant anatomical risk factors.

Data from the included studies suggest that the rate of recurrence might not be directly associated with joint function [41]. Long-term subjective and functional results of conservatively managed patients following patellar dislocation are for the most part satisfactory [46]. Lampros et al. [47] report that studies with objective measures combined with psychological readiness and a comprehensive understanding of the individual's specific tasks should be considered when assessing the ability to safely and successfully return to sport and, to a lesser extent, to daily life. Therefore, the level of functional demand is discussed to reflect on rehabilitation when working with non-operative and operative management of patellar instability.

The present study compared investigations in which the only therapeutic interventions were exercises. Smith et al. [40] compared the functional result of muscle strengthening of the vastus medialis obliquus with a general strengthening of the quadriceps muscle. The statistical difference in the Lysholm knee score and in the Tegner score between the groups at 12 months of intervention was not clinically relevant: isolated muscle training provided the same result as what obtained exercising the whole quadriceps. Although strengthening the quadriceps muscle and vastus medialis obliquus is the primary and main treatment advocated by many authors, the production of force in the knee extensors, hip abductors and hip extensor musculature is also an important target for rehabilitation [48], in addition to soft tissue flexibility [49]. Rood et al. [21] compared function using the Lysholm knee score and the rate of dislocation by performing two types of conservative intervention: taping and immobilization. They evaluated the outcomes of this regimen at 6 and 12 weeks, 1 year and 5 years. Taping resulted in higher values in terms of knee function compared to plaster immobilization, both in the short- and medium-term, with no difference in re-dislocation rate. Also, taping produced less muscular hypotrophy. Controlled mobilizations can be performed in the post-injury period to avoid loss of mobility and, in the future, muscle atrophy. Most of the surgeons recommend weight-bearing to tolerance and a knee brace during the first four weeks, with a range of motion from full extension to 30° of flexion during the first 15 days and up to 60° of flexion for an additional 15 days [50]. This study was corroborated by the use of a stabilizing and restrictive brace for 4 weeks after traumatic patellar dislocation for the first time, not resulting in a reduction in re-dislocations compared to the use of a brace without neoprene. Knee immobilization was associated with quadriceps muscle atrophy, more restricted knee ROM and worse functional outcomes in the first 6 months after injury [42]. In summary, although some studies have already addressed this issue, there is still no consensus on the ideal conservative treatment for primary patellar dislocation. Exercises to strengthen the quadriceps (including vastus medialis) and hip muscles; gain/maintenance of knee flexibility (hamstring stretching); use of braces with controlled free motion seem to be equivalent [38]. Surgical intervention is an appropriate option if patients continue to experience recurrent patellar dislocations and remain symptomatic, and conservative treatment options have been exhausted [51].

Some points are important to consider in this systematic review. The different methods used between the studies and the lack of randomised controlled trials represent important limitations of the present study. The criteria to diagnose instability and dislocation were not always clear in all studies, with different criteria used. Consequently, the different treatments used, whether surgical or conservative, influence the non-standardization of results and their heterogeneity. This also contributed to the fact that it was not possible to perform a meta-analysis. Follow-up studies may be more reliable for assessing instability and function. Postoperative rehabilitation was not the objective of our study and would need to be better described, as it is also part of the success of surgical treatment. Studies with better methodological controls and larger samples are important for greater study validity.

Conservative interventions were often biased, lacking in description or not reporting exactly the type, duration and structure of the physical sessions, which limit translation into the clinical practice. Further high-quality investigations are strongly required to establish the proper indications and efficacy of a structured rehabilitation program.

## Conclusion

Conservative treatment resulted in higher rates of recurrence of patellar dislocation compared to surgery. When comparing conservative treatments, the exercises were not well described, but exercises for the entire lower limb have effects similar to those concentrating on specific muscles, and the use of braces with controlled motion in the post-injury period is better than immobilization. For future interventions, it is important to consider conservative management before surgical treatment, when current active rehabilitation programs should be the basis of physical therapy intervention. However, it is essential to assess the level of functional demand of patients to tailor the treatment most appropriate to them.

## Data Availability

All data generated or analysed during this study are included in this published article.

## Abbreviations

Mesh: Medical Subject Headings
TT: Tibial tubercle
TG: Trochlear groove
PRISMA: Preferred Reporting Items For Systematic Reviews And Meta-Analyses
PISS: Patellar Instability Severity Score
BHS: Beighton Hypermobility Score
PROMS: Patient Reported Outcome Measures
KOOS: Knee Injury And Osteoarthritis Outcome Score
VMO: Vastus Medialis Oblique
VAS: Visual Analog Scale
N/A: Not applicable
RCT: Randomized controlled trials
